# Fostering clinical reasoning ability in preclinical students through an illness script worksheet approach in flipped learning: a quasi-experimental study

**DOI:** 10.1186/s12909-024-05614-9

**Published:** 2024-06-13

**Authors:** Jihyun Si

**Affiliations:** Department of Medical Education, Dong-A University College of Medicine, 32 Daesingongwon-ro, Seo-gu, Busan, 49201 Korea

**Keywords:** Illness script worksheet, Clinical reasoning, Flipped learning, Teaching method, Preclinical

## Abstract

**Background:**

The consensus that clinical reasoning should be explicitly addressed throughout medical training is increasing; however, studies on specific teaching methods, particularly, for preclinical students, are lacking. This study investigated the effects of an illness script worksheet approach in flipped learning on the development of clinical reasoning abilities in preclinical students. It also explored whether the impact of this intervention differed depending on clinical reasoning ability after dividing the students into high and low groups based on their pre-diagnostic thinking inventory (DTI) scores.

**Methods:**

This study used a one-group pre-post test design and convenience sampling. Forty-two second-year medical students were invited to participate in this study. The course, “clinical reasoning method,” was redesigned as an illness script worksheet approach in flipped learning. The course was an eight-week long program. The students met once or twice per week with a different professor each time and engaged with 15 clinical cases in small groups in one classroom. Each time, one professor facilitated seven groups in a single classroom. The effectiveness of the intervention was measured using DTI before and after the intervention. A learning experience survey was conducted with post-DTI assessment.

**Results:**

Thirty-six students participated in the survey and their data were analyzed. The mean pre-DTI score was 170.4, and the mean post-DTI score was 185.2, indicating an 8.68% increase (*p* < .001). Significant differences were also found in both high and low groups between the pre- and post-DTI assessments. However, the low group improved much more than the high group and exhibited a significant increase in one of the DTI subscales as well. The overall average score on the learning experience survey was 3.11 out of 4.

**Conclusion:**

The findings indicated that the intervention was an effective instructional method for the development of clinical reasoning in preclinical students and was more beneficial for students with a low level of clinical reasoning ability. This study demonstrated that the intervention can be a feasible and scalable method to effectively and efficiently train clinical reasoning in preclinical students in a classroom.

**Supplementary Information:**

The online version contains supplementary material available at 10.1186/s12909-024-05614-9.

## Introduction

The development of students’ clinical reasoning ability is an essential goal at every stage of medical education [1–4]. Traditionally, clinical reasoning has been considered to be implicitly learned by applying medical knowledge to patients’ situations [1, 4]. This learning during clinical clerkship is largely learning-by-doing; thus, opportunities to critically reflect on their performance are limited [5]. Furthermore, the limited number of patients available for practices during clinical clerkship, along with irregular and inconsistent feedback from experts, are major limitations [5, 6]. These challenges have raised concerns about whether medical students are adequately developing their clinical reasoning abilities during their clinical clerkship. Consequently, there is an increasing demand for explicit and direct instruction in clinical reasoning, particularly, for preclinical medical students as a direct preparation for clinical clerkship [2, 5, 7, 8].

The illness script refers to an organized knowledge structure that largely includes enabling conditions, pathophysiology, signs and symptoms [9, 10]. Experienced doctors have a repertory of illness scripts developed through the accumulation of clinical and biomedical knowledge and experience [3, 9–11]. When expert clinicians see patients, they make a diagnosis by activating relevant illness scripts, and gathering, analyzing, and evaluating patient information based on them [2, 6, 7]. Conversely, novice learners generally have structured knowledge according to the components of the curriculum, and often use hypotheses-deductive reasoning [2, 3]. They attempt to verify one hypothesis at a time in a clinical reasoning situation [10].

This script theory raises educational issues concerning how to train novice learners to restructure their learned medical knowledge in the form of illness scripts and make them infer like experts [9]. Illness scripts are key determinants of clinical reasoning performance in clinical settings, and they refine themselves based on their application to real cases [9, 11]. Typical cases involve the construction of default illness scripts that form the basis for further development [9, 11]. Therefore, deliberate practice for basic script building with clinical cases should focus on a steady trajectory toward expert-like clinical reasoning. However, specific instructional methods, particularly for preclinical student, are lacking.

Clinical reasoning based on real-life authentic clinical cases is a challenging task for preclinical students [12]. Levin et al. [8] developed an illness script worksheet approach as structured guidance for clinical reasoning training in preclinical students. This worksheet, which includes a clinical case vignette, illness scripts construction of three possible differential diagnoses, comparison and contrast of illness scripts, and a lab list to verify the diagnosis, serves as scaffolding for novice learners, guiding them through complex clinical reasoning processing. Scaffolding refers to the support and guidance that enables students to achieve a learning goal that is not achievable without them and is diminished when they are no longer needed [13]. Levin et al. [8] employed this worksheet with year two medical students to teach clinical reasoning and reported positive responses from students. Keeming et al. [7] and Moghadami et al. [2] also employed this illness script worksheet approach with undergraduate medical students and reported positive results in terms of clinical reasoning development.

While the previous studies demonstrated that the illness script worksheet approach can be an effective instructional strategy, they used the worksheet for experiments in the form of workshops or relatively short period [2, 7, 8]. To be a feasible method and give many iterations of practice in pre-clinical classrooms, the instructor workload must not be onerous and the feedback process must be efficient. This worksheet can serve not only as scaffolding to guide students’ learning processes, but also as cognitive feedback when presented in a completed form by professors. Cognitive feedback is feedback that helps learners reflect on the quality of their work, ultimately leading to its elaboration [14]. By comparing their worksheet with the expert-completed worksheet, students receive feedback on the gap between their performance and expert performance in key components of clinical reasoning processes. This enables students to construct more elaborate illness scripts and ultimately enhances their clinical reasoning skills. However, this discussion is lacking in the previous studies.

Small-group discussion-based activities based on clinical cases are considered to be effective instructional strategies for developing clinical reasoning in undergraduate education [1, 15]. Flipped learning, in which students learn foundational concepts about the case as required homework before class, and then use this knowledge to engage in clinical reasoning processes in small group settings during class time can be an effective instructional model for clinical reasoning development [15–18]. Adopting a flipped instructional model can accommodated small-group discussion-based activities by shifting didactic content delivery outside of class time, thus making time for case-based learning in class. Flipped learning is widely used in the field of health science and is known to increase students’ motivation, enhance their level of engagement, and interest in the subject, and foster critical thinking [16, 17]. Although flipped learning is a promising instructional model, relying solely on flipped learning based on clinical cases may not be sufficient for pre-clinical students with limited clinical experience to develop their clinical reasoning skills. There is a need for further instructional strategy that explicitly guides and nurtures the development of illness scripts, and facilitates expert-like clinical reasoning. Therefore, the illness script worksheet approach in a flipped learning setting seems to be an effective and efficient strategy to help preclinical students construct basic illness scripts and acquire clinical reasoning ability based on them. Furthermore, this structured format can make students less dependent on professors in small group discussions, allowing for many iterations as one facilitator can manage multiple groups in one classroom unlike problem-based learning (PBL). Thus, this intervention can be an effective and efficient strategy for preclinical students, but this issue has not been sufficiently discussed previously.

Thus, this study investigated the effects of the illness script worksheet approach in flipped learning on the development of clinical reasoning abilities among preclinical medical students. The illness script worksheet in this study was used to guide students’ clinical reasoning process and provide cognitive feedback from professors. The intervention was designed with one professor facilitating multiple groups in one classroom. The effectiveness of this intervention was measured using the diagnostic thinking inventory (DTI) before and after the intervention. DTI has been demonstrated as a reliable and valid assessment tool for clinical reasoning ability in various health care contexts [19–23]. It also examined whether the impact of this intervention differed depending on clinical reasoning ability after dividing the students into high and low groups based on their pre-DTI scores. Lastly, students’ learning experience were investigated through a survey to gather feedback on the intervention.

## Methods

### Participants and procedures

This study was conducted at a medical school in Korea and was approved by the Dong-A Institutional Review Board (2-1040709-AB-N-01-202109-HR-070-04). This study used a one-group pre-posttest design, and participants were drawn from convenience sampling. Forty-two second-year medical students enrolled in the course, “Clinical reasoning method” were all invited to participate in this study. Medical schools in Korea have six-year programs (two-year pre-medical and four-year medical programs including two-year clinical clerkships). The first two-year curriculum of the medical program consists of integrated blocks of biomedical and clinical knowledge. The participants were in their final quarter of the second year, with only one quarter remaining before entering their clinical clerkship. The pre- and post-DTI assessments were administered online for two weeks before the class started and after the class ended. In addition, a learning experience survey was conducted with the post-DTI assessment to explore students’ learning experiences. Online informed consent was obtained from all participants before the pre-and post-DTI assessment.

### Illness script worksheet

The illness script worksheets were modified from the one developed by Levin et al. [8] The illness script worksheet was composed of (1) a clinical case vignette, (2) its initial problem representation, (3) three possible differential diagnoses and their corresponding illness script constructions, (4) comparison and contrast of the illness scripts with the initial problem representation, and (5) development of a list of tests and their justification (Appendix 1). A clinical case vignette was presented in a format that reflects the way patients present in a doctor’s office, and then, the students were asked to make an initial problem representation, using semantic qualifiers. Thereafter, they were asked to provide the three most likely diagnoses for the case, starting from the highest likelihood, and fill in the illness script tables, including enabling conditions, pathophysiology, and consequences (signs and symptoms). They were then asked to compare and contrast the illness scripts with the initial problem representation, highlighting their similarities and differences. Finally, they were asked to develop a list of labs, tests, and imaging, and to justify how the suggested tests helped rule in/out each diagnosis. The students worked in groups and completed the worksheet as a group.

Levin et al. [8] created two versions (for students and facilitators) of the worksheet. In this study, the facilitators’ version was used as it included the comparison and contrast of illness scripts with the initial problem representation section, which was missing from the student version but was essential for clinical reasoning processes. Unlike Levin et al. [8], the students were asked to list three differential diagnoses in order, starting with the most likely one in the illness script tables. The illness script worksheet was presented to the students three times. First, it was presented in a completed form during the orientation session as an example and during class, it was presented as a guiding tool(scaffolding) for group work. Lastly, during the mini-lecture following group presentation, the professor’s completed worksheet was presented to students for cognitive feedback. This allowed students to reflect on and compare their works with expert-completed ones.

### Course design

The course, “Clinical reasoning method”, was transitioned from a traditional lecture-oriented model to a flipped-classroom model to accommodate illness script worksheet activities. It was an eight-week long program with 17 classes, each lasting two hours. The students met once or twice per week with a different professor each time and worked with 15 clinical cases (e.g. jaundice, hypotension, abdominal, pain and diarrhea) (Appendix 2). They worked in small groups, with six students per group, in one classroom, and the group remained unchanged. The first and last classes were the orientation and test sessions, respectively. Each time, one professor who developed a clinical case facilitated seven groups in a single classroom.

**Table 1:** Professors’ and students’ activities in flipped learning

	Professor	Students
Before class	∙ Develop a clinical case. ∙ Upload reading materials one week before a class on a learning management system (LMS) ∙ Develop and upload quizzes on the LMS	∙ Do the assigned reading
In class (95 min)	∙ Present online quizzes (around three) and check the answers (5 min). ∙ Hand out illness scrip worksheets to the group. ∙ Facilitate group discussions (50 min). ∙ Provide feedback on group presentation (40 min)	∙ Take quizzes ∙ Complete an illness script worksheet as a group ∙ Deliver a group presentation
Wrap-up (15 min)	∙ Deliver a mini-lecture (10 min) ∙ Take Q & A session (5 min)	∙ Q & A session ∙ Write reflective journals after each class

The structure of flipped learning, like other flipped learning, consists of before-class activities, class activities, and a wrap-up. Table 1 shows the professors’ and students’ activities in flipped learning. As shown in Table 1, the professors first developed a clinical case and uploaded assigned learning materials related to the clinical case to be discussed in class one week before a class online on the learning management system (LMS). They developed around three quizzes to check whether the students finished the assigned reading, and were ultimately prepared for class discussion. During the class, the professor let them take quizzes online through the LMS, then checked the answers as a class, and explained what the students did not understand. The professor then handed out the illness script worksheet to each group, and the students completed the worksheet through group discussions. An internet search was allowed during the discussion. While the students were discussing in a group, the professor went around the classroom and played as the facilitator role. They encouraged group discussions and answered the question as content experts. Each group then presented their completed worksheet in front of the class and the professor gave them feedback. Finally, the professor delivered a mini-lecture with the illness script worksheet completed by the professor to provide cognitive feedback by comparison. Finally, they had a Q&A session, and after the class the students wrote reflective journals regrading what they had learned, realized, or felt through their learning experience.

### DTI

DTI, developed by Bordge et al. [20], consists of 41 questions. It was designed to measure self-assessed clinical reasoning ability and has two subscales; flexibility in thinking (FT, 21 items) and evidence of structure in memory independent of content (SM, 20 items). FT involves using multiple approaches to explore diagnostic possibilities based on key patient interview features or general inquiries when forceful features do not arise yet. In addition, SM refers to the availability and accessibility of organized knowledge stored in memory during clinical reasoning [20]. DTI is based on illness script theory, focusing on the organization and availability of medical knowledge stored in memory as the prime determinant of diagnostic thinking [20, 21]. Previous studies have demonstrated that this is a reliable and valid assessment tool for clinical reasoning ability [19–24]. Each item of DTI contains a stem followed by two semantically opposing statement (e.g. “When I am interviewing a patient, I often seem to get one idea stuck in my mind about what might be wrong, or I usually find it easy to explore various possible diagnosis) and the students indicate where they fall most often when they deal with cases on a 6-point Likert scale. Each item was scored based on the proximity of the response most closely associated with expert diagnostic thinking. Higher subcategories or overall DTI scores indicate a more advanced level of diagnostic reasoning, with a maximum of 246 for the total score, 126 for FT and 120 for SM [20].

### Learning experience survey

A learning experience survey was administered to investigate the learners’ experiences during the newly developed course. The survey was adapted from a course evaluation survey conducted at the institution where this study was conducted, and it consisted of 11 questions on a 4-point Likert scale, including two open-ended questions regarding the strengths, and weaknesses of the course (Appendix 3).

### Data analysis

The collected data were analyzed using the IBM SPSS 27. The reliability of the survey items was assessed using Cronbach’s alpha. Changes in learners’ DTI and subcategory scores before and after the intervention were examined using a dependent t-test. To examine the impact of this intervention based on students’ clinical reasoning ability, they were divided into high and low groups according to their pre-DTI scores, and group comparisons were conducted using an independent t-test. The significance level for this study was set at *p* < .05. The survey on learning experiences was analyzed using descriptive statistics and data from the open-ended questions were analyzed and categorized through content analysis.

## Results

Among the 42 students enrolled to the course, 36 participated in both pre- and post- DTI assessments, and their data were analyzed. The respondent rate for the pre-DTI was 41 (98%) and the post- DTI was 36(86%); the number of female students was 15(41.7%), and male students was 21(58.3%), and the age range was 22–27(M(SD): 24.28, (1.09)). Reliability of total DTI items was 0.85 (Cronbach’s alpha); 0.66 and 0.83 for FT and SM respectively. These levels were acceptable [25].

**Table 2:** Descriptive statistics of the DTI assessments

	M(SD) *N* = 36	High group M(SD) *N* = 17	Low group M(SD) *N* = 19
Pre-DTI scores			
Total score	170.42(11.26)	179.71(8.89)	162.11(4.70)
FT	86.86(6.12)	91.12(6.02)	83.05(2.86)
SM	87.39(7.24)	92.88(5.36)	82.47(4.73)
Post-DTI score			
Total score	185.22(15.67)	189.76(11.07)	181.16(18.21)
FT	91.56(7.97)	93.76(6.06)	89.58(2.08)
SM	88.64(8.93)	91.00(7.15)	86.53(9.98)
Changes (post-DTI-pre-DTI scores)			
Total score		10.06(10.44)	19.05(17.45)
FT		2.65(5.53)	6.53(8.55)
SM		-1.88(8.08)	4.05(9.64)

FT; flexibility in thinking, SM: evidence of structure in memory

**Fig. 1:**
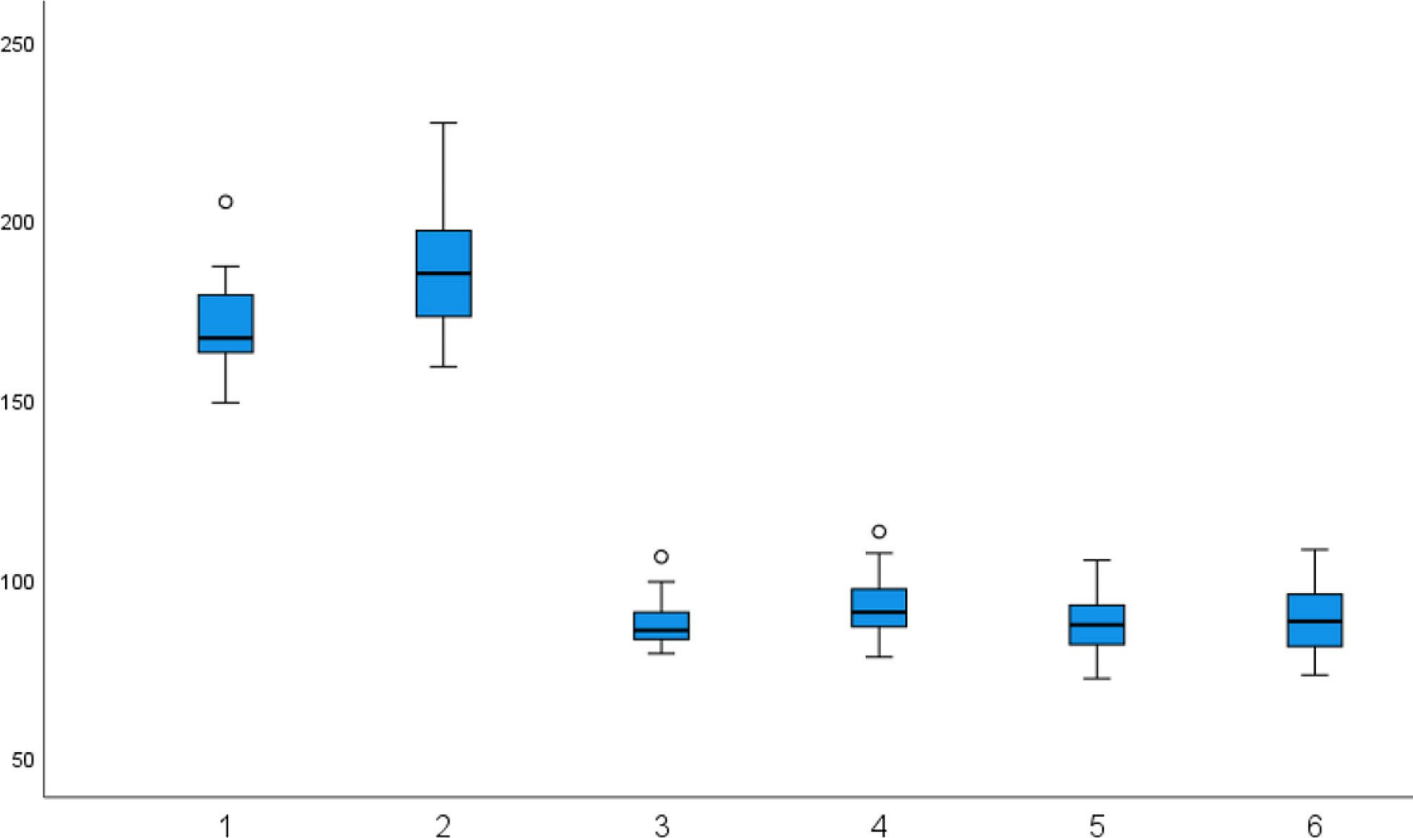
DTI scores. 1. Pre-DTI score, 2. Post-DTI score, 3. Pre-FT score, 4. Post- FT score, 5. Pre-SM score, 6. Post-SM score

The descriptive statistics of the DTI assessment are presented in Table 2; Fig. 1. There were significant statistical differences between the pre- and post-assessment in the total DTI and FT, revealing a high level of effect size in the total DTI, t (35) = 5.89, *p* < .001, d = 0.98, and a moderate level of effect size in the FT, t (35) = 3.78, *p* = .001, d = 0.63. However, there was no significant difference in the SM, t (35) = 0.81, *p* = .43 d = 0.13. When the students were divided into high and low groups according to their pre-DTI assessment, in the high group, there was a statistically significant difference between the pre- and post-DTI assessments, t (16) = 3.97, *p* = .001, d = 0.96, revealing a high level of effect size, but there were no significant differences in the FT, t (16) = 1.97, *p* = .07, d = 0.48, and the SM, t(16) = − 0.96, *p* = .35, d = − 0.23. However, in the low group, there were significant differences in the total DTI score, t (18) = 4.76, *p* < .001, d = 1.09, revealing a high level of effect size, and the FT, t (18) = 3.33, *p* = .004, d = 0.76, revealing a moderate level of effect size, but there was no significant difference in the SM, t (18) = 1.83, *p* = .083, d = 0.42. The results of independent t-tests to compare the changes in DTI scores between the high and low groups showed that there were no significant differences in the total DTI score, t (34) = -1.85, *p* = .073, d = − 0.62, the FM, t (34) = -1.59, *p* = .120, d = − 0.53 and the SM, t (34) = -1.99, *p* = .055, d = − 0.66.

### Learning experience survey

The reliability of the survey items was 0.87 (Cronbach’s alpha). Their descriptive statistics are presented in Table 3. The overall average score was 3.11 (4-likert scale). Item 7 had the highest score and item 8 showed the lowest score. That is, the students actively participated in learning activities in class and felt that the process of filling out the illness script and making clinical reasoning was not that difficult. In response to the open-ended questions, many students pointed out as its strengths, learning from peers during group activities, and improvements in clinical reasoning. They mentioned that “I found diverse perspectives through discussion”, “Peers explained what I did not know in group discussion,” “The process of discussion with my peers was really a good way to study,” and “I really improved my reasoning skills by doing this,” “It was very helpful to think about differential diagnosis and fill out the worksheet.” They also indicated as its strengths, learning various clinical cases, acquisition of clinical knowledge, reviews of previous learning, and feedback from instructors. Regarding weaknesses, students mentioned time constraints, frequency of classes, too many assigned reading materials and their delayed upload, the requirement to write reflective journals for each session, and insufficient medical knowledge. Students mentioned that “it was tight to do it in two hours,” “I was so busy. If more time had been given, a fuller discussion would have been taken place,” “Twice a week was too hard,” and “the requirement to write a reflective journal each time was burdensome.”

**Table 3:** Descriptive statistical results of the learning experience survey

	Items (1: strongly disagree, 4: strongly agree)	M(SD)
1	The course was designed to align with its learning objective of developing clinical reasoning abilities.	3.22(0.16)
2	I always attended the class after studying the assigned reading material before class.	3.50(0.12)
3	The difficulty level and quantity of the clinical cases utilized in the course were appropriate.	3.03(0.15)
4	In small group setting, collaborative learning with group members was effective.	3.39(0.13)
5	The teaching method was effective in developing clinical reasoning.	3.03(0.15)
6	Through reflective journal writing, I could monitor my progress and ascertain whether I was moving to the desired direction on my learning journey.	2.72(0.18)
7	I actively participated in learning activities in class.	3.83(0.06)
8	How was the process of filling out the illness script worksheet and making clinical reasoning? (1: Very difficult, 4: Not difficult at all).	2.50(0.15)
9	Overall, I am satisfied with this course.	2.75(0.15)
	Total	3.11(0.60)

## Discussion and conclusion

To develop the clinical reasoning ability of preclinical medical students, the illness script worksheet approach in a flipped learning setting was developed, and its effects were examined based on the changes in DTI scores before and after the intervention. According to the findings, the total DTI and FT scores improved significantly. This result indicated that the intervention was an effective method for developing the clinical reasoning abilities of preclinical medical students. The relatively high score (3.03/4) for the learning experience survey question on whether the intervention was effective in developing clinical reasoning supported this finding. This finding aligns with those of Levin et al. [8], Keeming et al. [7] and Moghadami et al. [2]. They all incorporated the illness script worksheet approach in a small group setting for undergraduate students and reported students’ positive responses [8], richer illness script descriptions, and better diagnostic performance [7], and significantly higher knowledge and script concordance test scores, and positive student responses than the control group [2].

In both the high and low groups, the post-total DTI scores significantly improved. In the subscales, only the low group demonstrated a significant increase in FT. In addition, the high group exhibited an average increase of 10.06 points in post-total DTI scores, whereas the low group showed an increase of 19.5 points. The comparison of these changes between groups did not reach statistical significance. However, the low group improved much more than the high group, and the changes in SM between two groups were almost the significant level (*p* = .055). Thus, it is fair to say that the intervention was an effective teaching method for both groups, but it was more beneficial for the low group than the high group. This lack of significance could be owing to the relatively small number of the subjects; however, further research is needed for a more accurate understanding.

In addition, the intervention seems an efficient format with which to rapidly improve FM in preclinical students. FM refers to the use of diversity of thinking methods or processes that can be applied during clinical reasoning (e.g., once I have made my mind about a patient, I am prepared to change my mind or I really do not like to change my mind) [20]. Collaborative learning during worksheet completion, and cognitive feedback through the completed worksheet by professors in flipped learning seem to have contributed to this finding. On the other hand, significant statistical differences were not found in SM between the pre-and post-assessments. SM refers to the availability of the knowledge structure in memory during clinical reasoning processes (e.g., when I know very little about a condition, I can still come up with a diagnosis, or I have great difficulty in reaching a diagnosis) [20]. One possible explanation for this insignificance might be the time constraint. Many students indicated time constraints as one of the weaknesses. One student stated, “I was so busy. If more time had been given, a fuller discussion would have been taken place.” Thus, if they had sufficient time to discuss and reorganize their thought, SM may have improved more. However, further research exploring the issue is necessary.

Overall students’ responses on the learning experiences were positive. They seemed to think that this intervention was appropriate for clinical reasoning development, induced active participation, and that collaborative learning was effective in class. In addition, they felt that constructing illness scripts and making clinical inferences based on them was not very difficult. Thus, in general, working with peers using a guided scaffold (the illness script worksheet) in flipped learning seemed to work as expected. However, many students identified time constraints as a weakness as mentioned above. This course lasted two hours, and it turned out that they wanted more than two hours for discussion. Excessive pre-class reading materials and their delayed upload, class frequency (twice per week), and the requirement to write reflective journals for each session were also noted as weaknesses. These issues need to be adjusted according to the contexts to improve student satisfaction.

An explicit clinical reasoning development curriculum should be integrated at every stage of medical trainings, and then, teaching strategies should be tailored appropriately for students at different stages of medical training [1, 4, 5]. Based on the findings, the illness script worksheet approach, which explicitly and directly guided pre-clinical students throughout clinical reasoning processes, provided appropriate support in classroom settings. The small group setting in flipped learning also diminished the workload associated with resolving clinical cases by distributing it among group members [12]. Additionally, unlike PBL, which typically requires one facilitator per group in medical education contexts, this intervention demonstrated the feasibility of one facilitator in a classroom managing multiple groups and providing sufficient feedback regarding the clinical reasoning process. Thus, this study demonstrated that the intervention can be a feasible and scalable method to effectively and efficiently train clinical reasoning in preclinical students in classrooms. Furthermore, while this intervention was implemented in a flipped learning setting for this study, its effective application in fostering clinical reasoning can be extended to any small group setting with careful instructional design.

In addition, most of the previous research that explored the effectiveness of flipped learning in terms of clinical reasoning development was carried out in nursing and pharmacy, and evidence of the effectiveness of flipped learning in medical education is lacking [16, 26]. This study provided a positive evidence for medical education. One of the key features of successful flipped learning is an effective in-class learning design that encourages students to engage in collaborative knowledge application activities and properly guides this learning process [27, 28]. In this study, the illness script worksheet approach in flipped learning induced and guided collaborative knowledge application, ultimately resulting in positive results.

In conclusion, this study explored the effects of the illness script worksheet approach in flipped learning on the development of clinical reasoning in preclinical students. The findings showed that the intervention was an effective instructional method for the development of clinical reasoning in preclinical students and was more beneficial for students with a low level of clinical reasoning ability. Furthermore, the intervention proved to be an efficient format for rapidly improving flexibility in thinking in preclinical students. Ultimately, it demonstrated that this intervention is a feasible and scalable method to effectively and efficiently train preclinical students in classroom. A longitudinal study needs to be conducted to examine whether the effects of this intervention can be transferred to real clinical contexts.

This study has several limitations. First, this was a single-center study with a relatively small number of participants. This may limit the generalizability of the findings. Future studies with larger sample sizes and diverse learner backgrounds are necessary to confirm these findings. Second, DTI is a reliable and valid tool for assessing clinical reasoning ability. However, this tool has not been validated in the Korean context, which might have affected its reliability. Third, this study relied on survey data. When interpreting the results of this study, the potential bias towards positive responses should be considered. Future studies should incorporate behavioral data to compare with the results of this study.

### Electronic supplementary material

Below is the link to the electronic supplementary material.

Supplementary Material 1

## Data Availability

The datasets used during the current study are available from the corresponding author on reasonable request.

## Abbreviations

DTI: Dignostic thinking inventory
LMS: Learning management system
