# Supplementary material for: Fostering clinical reasoning ability in preclinical students through an illness script worksheet approach in flipped learning: a quasi-experimental study
Source: BMC Med Educ. 2024 Jun 13;24:658. doi: 10.1186/s12909-024-05614-9 (PMC11177456; doi:10.1186/s12909-024-05614-9)
Supplement: Supplementary file 1 — Supplementary Material 1 [file 12909_2024_5614_MOESM1_ESM.docx]

**Appendix 1.**

**Illness script worksheet**

Fill in the form as a group and present your choices to the class, justifying your choices.

1. In small groups, read the case and present a problem representation, highlighting key features.

**Case:**

**Initial Problem Representation:**

*Provide a short summary of the case, using semantic qualifiers (eg. Young/old, acute/chronic, diffuse/localized, mild/severe, etc.)*

2. In a small group discussion, present three possible differential diagnoses for the case and write illness scripts for each differential diagnosis. List them in order, starting with the most likely one.

| **Illness Scripts:** | *Diagnosis #1* | *Diagnosis #2* | *Diagnosis #3* |
| --- | --- | --- | --- |
| **Enabling conditions** |  |  |  |
| **Pathophysiology** |  |  |  |
| **Clinical**  **Consequences (signs and symptoms)** |  |  |  |

**3.** Compare and contrast the illness scripts with the initial problem representation, listing the similarities and differences of important features.

**4.** In small groups, develop a list of labs, tests, and imaging and justify how proposed tests will help rule in/out each diagnosis.

**Labs/Imaging:**

This worksheet was adapted from Levin M et al. MedEdPORTAL. 2016;12: 10445 and modified to suit the purpose of this study. Jihyun S. Korean J Med Educ. 2024;36(2):213-221.

**Appendix 2.**

**Clinical case example: Hypotension**

A 29-year-old female patient presents to the emergency room with hypotension as the chief complaint. She has no significant medical history and is currently at 23 weeks gestation as a primigravida. During a routine prenatal examination at the obstetrics and gynecology clinic, her blood pressure was found to be low, prompting referral to the hospital. She had been taking iron supplements since the onset of pregnancy, with no additional medications. Over the past week, she has experienced dizziness and palpitations upon standing up, occurring approximately 3-4 times a day, and occasionally felt chest tightness. While her blood pressure was a concern during the prenatal examination, in the emergency room, her blood pressure was measured at 85/50 mmHg, heart rate at 105 beats per minute, respiratory rate at 20 breaths per minute, and her temperature was normal. Bilateral pedal and ankle edema were observed on physical examination, with swelling starting approximately two weeks ago, causing discomfort when wearing shoes. Prenatal examination revealed no abnormalities in amniotic fluid volume or fetal status.

**Appendix 3.**

**The learning experience survey**

Please rate your level of agreement with the following statements:

|  | **1**  Strongly Disagree | **2**  Disagree | 3  Agree | 4  Strongly Agree |
| --- | --- | --- | --- | --- |
| 1. The course was designed to align with its learning objective of developing clinical reasoning abilities. |  |  |  |  |
| 2. I always attended the class after studying the assigned reading material before class. |  |  |  |  |
| 3. The difficulty level and quantity of the clinical cases utilized in the course were appropriate. |  |  |  |  |
| 4. In small group setting, collaborative learning with group members was effective. |  |  |  |  |
| 5. The teaching method was effective in developing clinical reasoning. |  |  |  |  |
| 6. Through reflective journal writing, I could monitor my progress and ascertain whether I was moving to the desired direction on my learning journey. |  |  |  |  |
| 7. I actively participated in learning activities in class. |  |  |  |  |
| 8. How was the process of filling out the illness script worksheet and making clinical reasoning? (1: Very difficult, 4: Not difficult at all). |  |  |  |  |
| 9. Overall, I am satisfied with this course. |  |  |  |  |

10. What did you think were the strengths of this class?

11. What did you think were some weaknesses of this class?

Thank you.
